# A Study to Enhance the Nitrate-Nitrogen Removal Rate without Dismantling the NF Module by Building a PFSA Ionomer-Coated NF Module

**DOI:** 10.3390/membranes11100769

**Published:** 2021-10-09

**Authors:** In-Kee Park, Jian Hou, Jaehan Yun, Hee-Dae Lee, Chang-Hyun Lee

**Affiliations:** 1 Department of Energy Engineering, Dankook University, Cheonan 31116, Korea; inkee0149@gmail.com (I.-K.P.); houjimmy@naver.com (J.H.); ruri7220@naver.com (J.Y.); 2 Coway R & D Center, Seoul 08826, Korea; lhd1371@coway.co.kr

**Keywords:** NF module, supercritical fluid, dispersion, PFSA ionomer, nitrate-nitrogen removal

## Abstract

Water resource pollution by nitrate-nitrogen, mainly caused by anthropogenic causes, induces eutrophication of water resources, and indicates the degree of organic pollution. Therefore, this study devised a method for coating PFSA ionomer with excellent chemical resistance without disassembling the module to improve the removal rate of nitrate-nitrogen in water by using a cyclic coating method on a commercially available nanofiltration membrane (NF membrane) module. Nafion was prepared as a supercritical fluid dispersion using a high-temperature and high-pressure reactor, and the particle size and the degree of dispersion of the dispersion were analyzed by DLS. The crystallinity was confirmed through XRD by drying the dispersion in the liquid state. After the dispersion was prepared as a membrane according to the heat treatment conditions, the characteristics according to the particle size were analyzed by tensile strength and TEM. The nitrate-nitrogen removal rate of the NF membrane module coated with the dispersion was increased by 93% compared to that before coating. Therefore, the result showed that the cycle coating method devised in this study could efficiently coat the already commercialized module and improve performance.

## 1. Introduction

Nitrogen is an essential component of plant and animal proteins and is a vital component of organisms but is toxic when contained in excessive amounts [1]. In particular, artificial contamination caused by fertilizer spraying and livestock wastewater is the leading cause [2], and it is the causative agent of methemoglobinemia (cyanosis) when infants ingest wastewater [3]. Nitrate-nitrogen indicates the degree of contamination according to the content of nitrogen compounds, such as nitrogen oxides and ammonia, in water or soil [4]. The concentration of nitrate-nitrogen is limited to 10 ppm or less based on water quality standards [5]. However, as a result of a 2004 World Health Organization (WHO) survey of 2000 of the world’s drinking water sources [6], 30% of them had a nitrate concentration of 24 mg/L (24 ppm) or higher, and the problem of nitrate-nitrogen continues to be an issue [6].

So far, the methods to remove nitrate-nitrogen in water include biological denitrification [7], ion exchange, reverse osmosis, etc., and membrane technology and electrodialysis methods are most commonly used [2]. The biological denitrification method uses a large amount of disinfectant, so there is a stability problem [8]. The ion exchange method is disadvantageous as divalent sulfate ions are preferentially removed before nitrate-nitrogen, producing regeneration waste [9]. It is not easy to dispose of the used regeneration waste since it contains a large amount of nitrate-nitrogen. Once released, it causes soil contamination and eutrophication of water quality [10]. The reverse osmosis method is used as the most effective method, together with the electrodialysis method [11]. Still, high pressure or additional electrical energy is required to treat the contaminated water, so the operation cost is considerable [9]. Therefore, the cycle-coating method used in this study has the advantage of being able to easily coat the commercially available NF membrane module (nanofiltration membrane module) without disassembling it and through this, a higher removal rate of nitrate-nitrogen can be expected.

A perfluorosulfonic acid ionomer (PFSA ionomer) consists of a polytetrafluoroethylene (PTFE) backbone having a chemically strong structure [12] and a perfluoro side chain containing a sulfonic acid group (-SO_3_H) at the end [13]. The perfluoro structure containing sulfonic acid induces a hydrophobic/hydrophilic microphase separation form for fast proton conduction [14]. The high electronegativity of the F atom makes it easy to release a proton from the sulfonic acid group [15]. It is expected that these PFSA ionomers will be coated on the NF membrane module in a dispersed form in an aliphatic alcohol-water mixture to prevent the permeation of nitrate-nitrogen. In addition, as PFSA ionomers have higher reliability than hydrocarbons in chemical and thermal stability, as demonstrated in several kinds of literature [16], Nafion dispersions (e.g., 5 wt% Nafion D521, DuPont, USA) are commonly used [17]. However, Nafion dispersions have difficulties controlling the particle size and dispersibility characteristics, and attempts have been made to control particle size or dispersibility through chemical and physical routes [18].

Supercritical fluids (SCFs) have fast heat and material movement, low viscosity, and high permeability into micropores due to their unique properties, such as high diffusion coefficients and other liquids and gases [19]. In addition, it is widely used in industry and research [20] because it can solve the technical problems of adverse effects on the environment or low efficiency in the existing reaction process and is particularly useful for the synthesis of unique pharmaceuticals, polymers, and nanomaterials [21]. Therefore, in this study, a Nafion ionomer dispersion having colloidal particles smaller than a commercially available Nafion dispersion obtained by treatment with water-soluble aliphatic alcohol studied in the past was prepared through supercritical technology. Then, we coated the Nafion ionomer dispersion on the surface of the separation membrane without separating the commercially available NF membrane module, using the cyclic coating method, and examined the nitrate-nitrogen removal properties in water.

## 2. Materials and Methods

### 2.1. Materials

1-propanol (NPA, >99.9%) and isopropyl alcohol (IPA, >99.7%) were purchased from Sigma Aldrich Co. (Saint Louis, MA, USA) and used without further purification. The sodium nitrate (NaNO_3_, >99.9%), calcium chloride dihydrate granular (CaCl_2_ 2H_2_O, >98%), and magnesium sulfate heptahydrate (MgSO_4_ 7H_2_O, >99%) were purchased by Daejung Chemical & Metal. Co. Ltd., Siheung-si, Korea. The NF membrane module (NF module 8 inch, NF8S) was provided by Coway Co., Ltd., Seoul, Korea. The deionized water (D.I. water) used to prepare and wash the dispersion was prepared using an ultrapure water manufacturing apparatus Milli-Q (Progard^®^ T3, Millipore, Darmstadt, Germany). Nafion 117 film (Dupont Co., Wilmington, NJ, USA) was used to prepare a PFSA ionomer dispersion for coating the NF membrane module.

### 2.2. PFSA Ionomer Dispersion Preparation and Analysis

**Figure 1:**
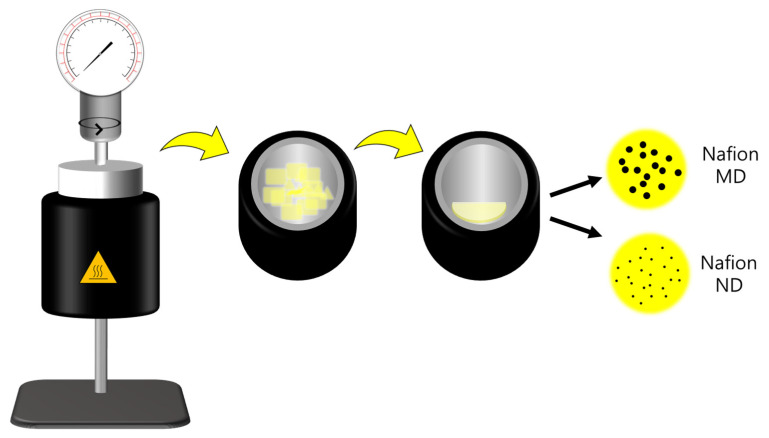
Preparation process schematic diagram of Nafion ionomer micro/nano-dispersions.

The PFSA ionomer dispersion used to prepare the coating layer of the NF module was made with Nafion117 as liquid SCFs through a supercritical dispersion process. To prepare Nafion117 as SCF, it was prepared by mixing NPA and D.I. water as solvents in a high-pressure/high-temperature reactor (4560 Mini-Bench Reactor System, PARR, Moline, IL, USA) at a volume ratio of 1:3 (Figure 1). The particle size of the PFSA ionomer dispersion prepared in a high-pressure/high-temperature reactor was confirmed using dynamic light scattering (DLS, Zetasizer Nano ZS, Malvern, Worcestershire, UK). Nafion micro-dispersion ionomer solution (Nafion MD) and Nafion nano-dispersion ionomer solution (Nafion ND) were named according to the dispersed particle size. After drying the prepared ionomer dispersion, crystallinity and D-spacing analysis were performed using an X-ray diffraction analyzer (Ultima IV, Tokyo, Japan). XRD analysis was measured at 5°/min in the 5–30° scan range at 35 mA and 40 kV, and the degree of crystallinity (X_c_) in the XRD diffraction pattern obtained through the analysis was calculated using the following Equations (1) and (2) [13]:

(1) $$\mathrm{Diffraction}\;\mathrm{vector}\;\left(q\right)=\frac{4\pi }{\lambda \sin\left(\theta /2\right)}$$

(2) $$\mathrm{Crystallinity}\;\left(X_{c}\right)=\frac{\int_{0}^{\infty }I_{c}\left(q\right)q^{2}dq}{\int_{0}^{\infty }[I_{c}\left(q\right)q^{2}+I_{a}\left(q\right)q^{2}]dq}$$

In Equation (1), *λ* represents the wavelength of Cu-K_a_ light used for analysis, and *I_a_*(*q*) and *I_c_*(*q*) represent the peak intensities of amorphous and crystalline wavelengths, respectively. The degree of crystallinity was expressed by dividing the crystal scattering area by the sum of the crystal and amorphous scattering areas. At this time, 2θ values corresponding to crystalline and amorphous in the XRD diffraction pattern of the PFSA ionomer dispersion are 16° and 17.5°, respectively.

### 2.3. NF Membrane Module Coating

**Figure 2:**
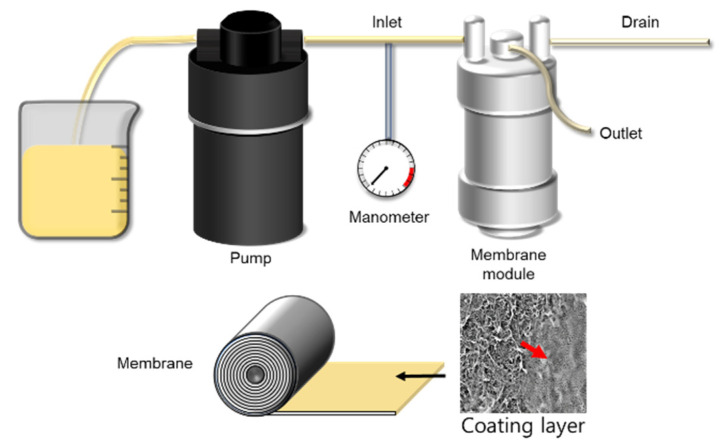
NF membrane module coating process via a simple circulation process.

As a coating solution for forming the PFSA ionomer layer on the NF membrane module, a PFSA ionomer dispersion was prepared at 5 wt.% using a solvent containing a 55:45 composition ratio of NPA and D.I. water. Then, after washing for 10 min with D.I. water through the inlet and drain of the membrane module, the coating solution was circulated at 100 mL/min for 30 min to coat and dried in an oven at 85 °C for three days (Figure 2). In order to measure the basic properties of the NF membrane coating layer, a membrane was prepared under similar conditions using PFSA ionomer dispersion. The membrane prepared under similar conditions was developed through solidification in a convection oven for 24 h at 85 °C, the same temperature condition applied to the preparation of the ionomer layer.

Small-angle X-ray scattering (SAXs, Model Bl 4C SAXs II, Pohang, Korea) was used to analyze the inter-domain distance, which means the average distance between the hydrophilic domains of the coating layer. The X-ray beam had power of 3.0 GeV, a wavelength of 0.07 nm, 1 × 1012 ph/sec, and a beam flux size of 100 (V) μm × 300 (H) μm, and the sample-detector distance (SDD) between the sample and the detector was set to 1 m. Here, the sample was stacked to a thickness of 100–120 μm and was sampled with an area of 1 × 1 cm^2^. The SAXs profile result was calculated using Bragg’s equation as in Equation (3), which corresponds to *q*(1)*max* [22]:

(3) $$D\;\left[Å\right]=\frac{2\pi }{q\left(1\right)max}\;$$

The cross-section of the NF membrane was measured at an acceleration voltage of 20 kV using a scanning electron microscope (SEM, Scanning Electron Microscope, JEOL, JSM-6360, Tokyo, Japan). The sample was attached to a double-sided carbon tape (Nishin EM, Tokyo, Japan) and coated with platinum (Pt) for pretreatment. In addition, the chemical composition of the sample was confirmed by energy-dispersive X-ray spectroscopy (EDS).

Additionally, to confirm the morphology of the membrane prepared from the ionomer dispersion, the sample was measured at an acceleration voltage of 120 kV using a transmission electron microscope (FE-TEM, Field-Emission Transmission Electron Microscopy, LIBRA 120, Carl Zeiss, Jena, Germany). The sample was sequentially pre-treated (fixation, dehydration, and embedding) using dimethylaminoethanol (DMAE), nonenyl succinic anhydride (NSA), diglycidyl ether polypropylene glycol (DER), and 4-vinylcyclohexene dioxide (VCD). The prepared sample was cut using an ultramicrotome (EMUC7, Leica, Hessen, Germany) and then immersed in lead citrate to stain the ionic domain of the sample, washed with D.I. water, and dried thoroughly before use.

### 2.4. Nitrate Removal Characteristics of Ionomer Coated Modules

A feed solution was prepared by mixing CaCl_2_-MgSO_4_ with 30 ppm NaNO_3_ so that the hardness became 600 ppm to observe the nitrate-nitrogen removal characteristics of the coated NF module. The prepared feed solution was passed through at a pressure of 3.54 kgf/cm^2^ at room temperature (25 °C), and the residual NaNO_3_ content was analyzed to show the removal rate.

## 3. Results

**Figure 3:**
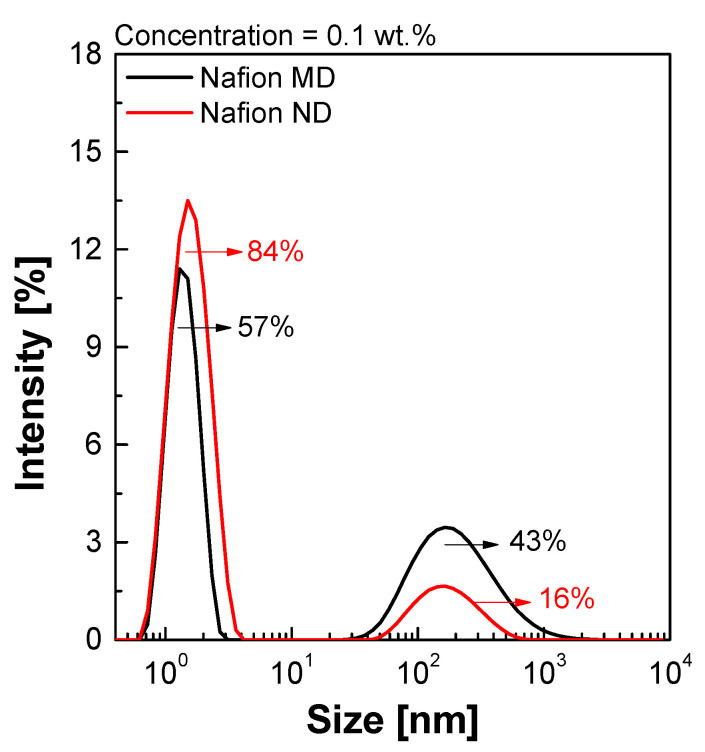
Particle size distribution pattern of Nafion ionomers by DLS.

Figure 3 shows the particle size distribution of the Nafion ionomer dispersion measured by DLS. Ionomer particles were classified into a small particle area (<10 nm), a medium particle area (10–100 nm), and a large particle area (100–10 µm), and Nafion MD and Nafion ND according to the change in the relative water content in the aqueous IPA mixture showed a bimodal ionomer particle distribution regardless of the E_W_ value. For the Z-average size of each dispersion, the measured size of Nafion ND was relatively smaller: 9.4 nm for Nafion MD and 1.76 nm for Nafion ND, because the Nafion MD small particle distribution is relatively low (57%) compared to the Nafion ND’s distribution (84%). Therefore, since the Nafion ND has smaller pores than the NF membrane with excellent SCF permeability, it seems to increase the nitrate-nitrogen removal rate more effectively when applied to the module coating with its higher permeability.

**Figure 4:**
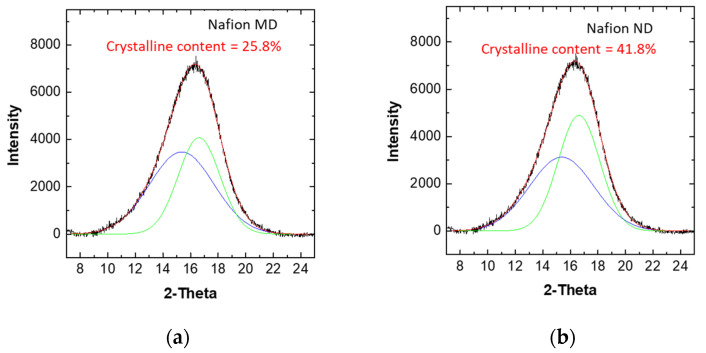
XRD pattern of solidified-state Nafion ionomers; (**a**) Nafion MD, (**b**) Nafion ND.

Figure 4a,b shows the results of measuring the crystallinity of Nafion MD and Nafion ND using XRD. Nafion ND and Nafion MD have the same molecular weight. Still, like Nafion ND, if the particles are entangled at the nano level, they will solidify, and the density of the polymer may increase. At the same time, the self-assembly between the hydrophilic functional group and the hydrophobic functional group improves the regularity of the polymer packing, thereby increasing the crystallinity [23]. Due to this effect, the crystallinity of Nafion ND was increased by 16% to 41.8%, compared to 25.8% of Nafion MD.

**Figure 5:**
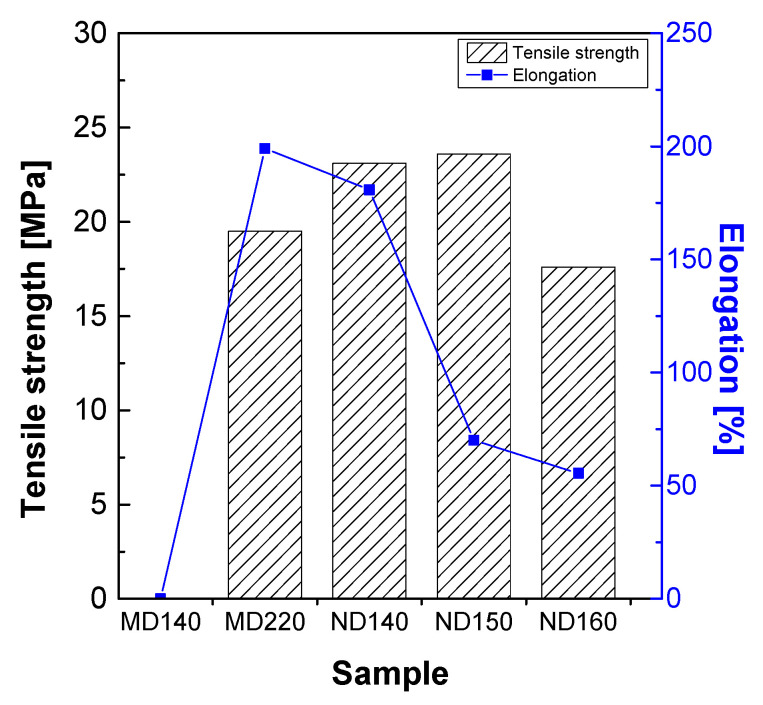
Mechanical property of solidified-state Nafion ionomers.

Figure 5 shows the results of analyzing the tensile strength and elongation of the membranes prepared according to the heat treatment temperature of Nafion MD and Nafion ND. The micro-dispersion membrane (MD) showed tensile strength and elongation that were impossible to analyze under the heat treatment conditions below 220 °C, and MD220 processed at 220 °C had a tensile strength of 19.5 MPa and an elongation of 199.1%. Additionally, unlike MD, the nano-dispersion membrane (ND) exhibited better tensile strength than MD220 at a relatively low temperature (140 °C). According to the particle size, the change of the heat treatment temperature is not smooth for heat transfer to the inside of the particle in the case of MD because the size of the particles included in the dispersion is relatively larger than that of ND. Therefore, it is considered that the intermolecular entanglement caused by the intermolecular interaction occurs at a higher temperature in the MD compared to the ND. From the XRD results, the higher crystallinity of ND than that of MD affected the increase in tensile strength [24]. However, since the size of the ND particles is small, the effect on temperature is significant. Therefore, when the ND was heat-treated at 160 °C or higher, the intermolecular bonding force due to deterioration should have decreased, which might cause lower tensile strength and elongation.

**Figure 6:**
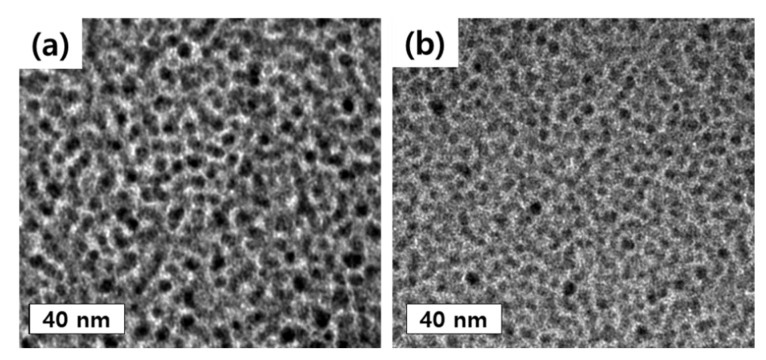
TEM images of solidified-state Nafion ionomers; (**a**) MD220, (**b**) ND140.

**Figure 7:**
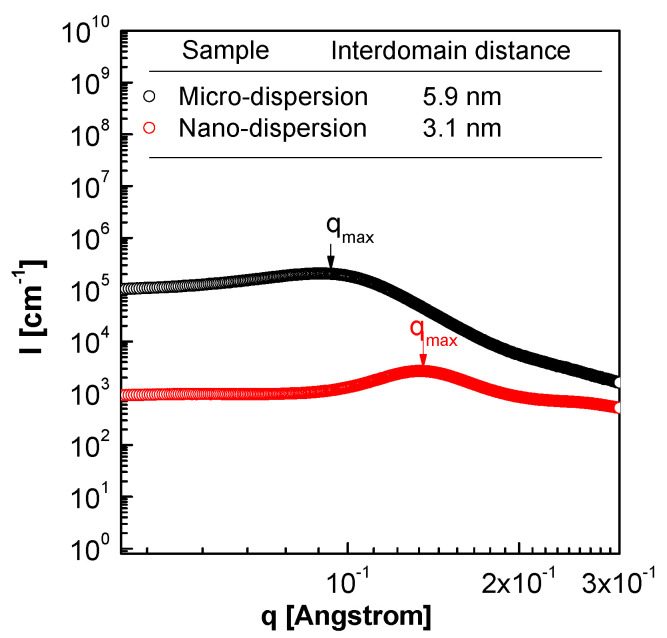
SAXS spectra of solidified-state Nafion ionomers.

Figure 6 shows the TEM images of MD220 and ND140 prepared with PFSA ionomer dispersion, and the samples were stained with lead citrate to understand the higher-resolution film morphology. Due to lead citrate, complex ions were formed in the mixture of sulfonic acid groups (-SO_3_), which causes Pb^2+^ ions contained in the colorant to bubble, and the hydrophilic domain (SO_3_H) appears black. Regardless of the manufacturing method, hydrophilic and hydrophobic regions were observed in all ionomer membranes. Through TEM, the size of the hydrophilic domains of the sulfonic acid groups of MD and ND were 6.5 and 2.8 nm, respectively, and this trend was similar to 5.9 and 3.1 nm in SAXS analysis, which can measure the distance between bulky hydrophilic domains (Figure 7). This meant that Nafion ND had a shorter ion pass way between hydrophilic domains than Nafion MD; consequently, the distance of the whole domains was shortened [13]. In the final NF membrane coating process, considering the ineffective influence on the coating inside of pores due to the larger particle size of Nafion MD. Therefore, Nafion ND was used that could effectively penetrate inside based on the small particle size.

**Figure 8:**
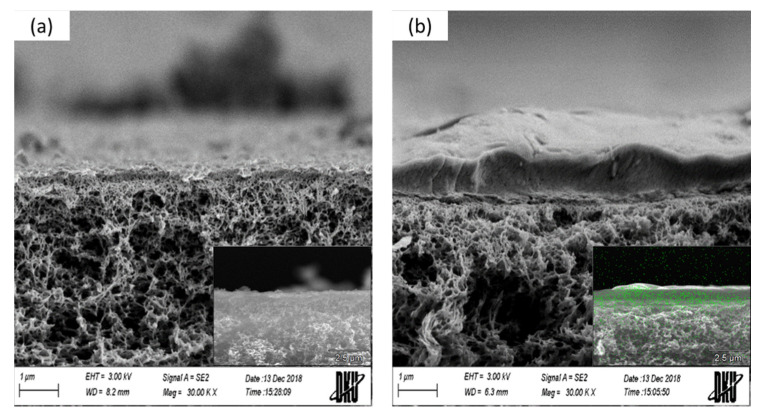
SEM cross-section images of NF membranes before/after ionomer coating. (**a**) NF8S_Ref-before, (**b**) NF8S_C-after and F-EDS.

Figure 8 shows the results of SEM and EDS analysis. In SEM images, the Nafion ND layer was formed on the surface of the NF membrane. Furthermore, F atoms were also partially confirmed inside the membrane and on the surface based on the EDS result. This result seems to be caused by the Nafion ND having a smaller particle size than the pores of the NF membrane, and the particles easily penetrated the membrane during the ionomer coating process, as shown in Figure 2. It is expected that nitrate-nitrogen will be effectively removed due to the penetration of ionomers into the pores, but a decrease in flux is expected.

**Figure 9:**
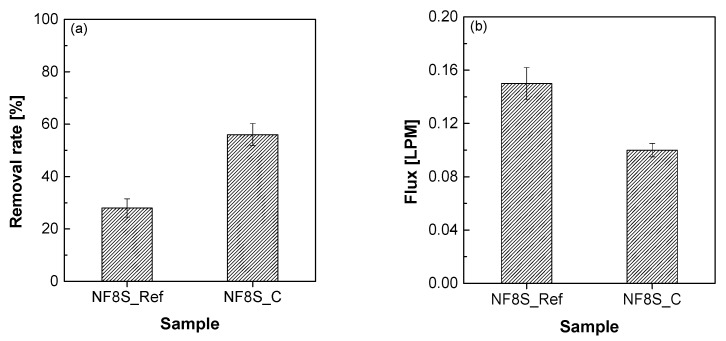
(**a**) Nitrogen nitrate removal rate, and (**b**) filtration flux.

**Figure 10:**
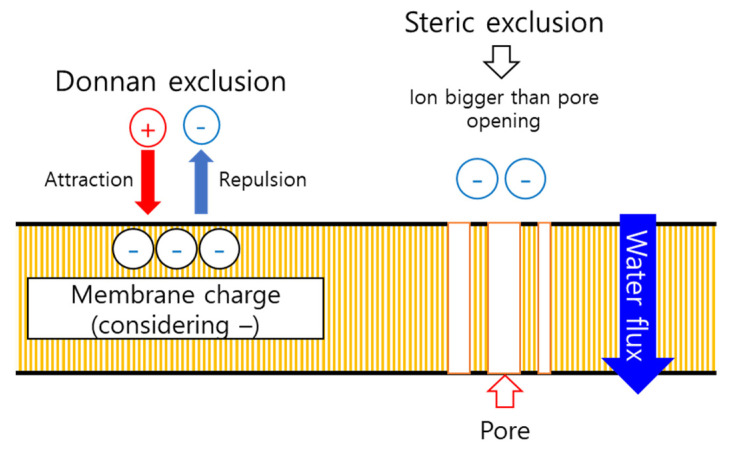
Synergetic effect diagram of ionomer coating on the removal rate.

Figure 9 shows the change in the nitrate-nitrogen removal rate and permeability according to the presence or absence of Nafion ND coating on the NF membrane module. Figure 10 shows the principle of the removal mechanism by this hybrid ionomer coating process. The removal rate of nitrate-nitrogen was increased by 93% through the ND ionomer coating, most possibly due to the effect of the smaller pore size of the NF membrane and removal due to the difference in charge between the membrane and ions (Figure 10) [25]. However, the permeability decreased by about 0.05 LPM due to the ionomer blocking the pores, as shown in Figure 6. Therefore, the permeability was slightly decreased by coating the ionomer, but the removal rate was improved. Coating the ionomer without separating the module increased the removal rate of nitrate-nitrogen contained in water.

## 4. Conclusions

This study analyzed a method for coating Nafion ionomers without disassembling the commercially available NF membrane module and the removal efficiency of nitrate-nitrogen in water. Nafion ionomers with strong chemical resistance could be dispersed in the form of SCFs using a high-pressure/high-temperature reactor. Then, the physical properties were analyzed according to the particle size of the dispersed ionomers. Changes in tensile strength and elongation were verified according to the heat treatment temperature, and excellent physical properties were identified even though ND140 was heat-treated at a lower temperature than MD220. In addition, changes in the physical properties proved that the Nafion ND had a higher degree of crystallinity than Nafion MD through XRD. Nafion ND was coated on the NF membrane module by a simple cyclic method. We confirmed, through SEM and EDS, the ionomer was coated on the module membrane, and the nitrate-nitrogen removal rate increased by 93%. The cyclic coating method conducted in this experiment was designed to coat the ionomer without disassembling the previously manufactured NF membrane module. Through this process, the removal rate of nitrate-nitrogen could be effectively increased.
